# Self-assembled peptide hydrogel loaded with functional peptide Dentonin accelerates vascularized bone tissue regeneration in critical-size bone defects

**DOI:** 10.1093/rb/rbae106

**Published:** 2024-08-23

**Authors:** Yijuan Liu, Li Li, Mengjiao He, Yanmei Xu, Zekai Wu, Xiongcheng Xu, Kai Luo, Hongbing Lv

**Affiliations:** Fujian Key Laboratory of Oral Diseases & Fujian Provincial Engineering Research Center of Oral Biomaterial & Stomatological Key Laboratory of Fujian College and University, School and Hospital of Stomatology, Fujian Medical University, Fuzhou 350002, People’s Republic of China; Institute of Stomatology & Laboratory of Oral Tissue Engineering, School and Hospital of Stomatology, Fujian Medical University, Fuzhou 350002, People’s Republic of China; Fujian Key Laboratory of Oral Diseases & Fujian Provincial Engineering Research Center of Oral Biomaterial & Stomatological Key Laboratory of Fujian College and University, School and Hospital of Stomatology, Fujian Medical University, Fuzhou 350002, People’s Republic of China; Institute of Stomatology & Laboratory of Oral Tissue Engineering, School and Hospital of Stomatology, Fujian Medical University, Fuzhou 350002, People’s Republic of China; Fujian Key Laboratory of Oral Diseases & Fujian Provincial Engineering Research Center of Oral Biomaterial & Stomatological Key Laboratory of Fujian College and University, School and Hospital of Stomatology, Fujian Medical University, Fuzhou 350002, People’s Republic of China; Institute of Stomatology & Laboratory of Oral Tissue Engineering, School and Hospital of Stomatology, Fujian Medical University, Fuzhou 350002, People’s Republic of China; Fujian Key Laboratory of Oral Diseases & Fujian Provincial Engineering Research Center of Oral Biomaterial & Stomatological Key Laboratory of Fujian College and University, School and Hospital of Stomatology, Fujian Medical University, Fuzhou 350002, People’s Republic of China; Institute of Stomatology & Laboratory of Oral Tissue Engineering, School and Hospital of Stomatology, Fujian Medical University, Fuzhou 350002, People’s Republic of China; Fujian Key Laboratory of Oral Diseases & Fujian Provincial Engineering Research Center of Oral Biomaterial & Stomatological Key Laboratory of Fujian College and University, School and Hospital of Stomatology, Fujian Medical University, Fuzhou 350002, People’s Republic of China; Institute of Stomatology & Laboratory of Oral Tissue Engineering, School and Hospital of Stomatology, Fujian Medical University, Fuzhou 350002, People’s Republic of China; Fujian Key Laboratory of Oral Diseases & Fujian Provincial Engineering Research Center of Oral Biomaterial & Stomatological Key Laboratory of Fujian College and University, School and Hospital of Stomatology, Fujian Medical University, Fuzhou 350002, People’s Republic of China; Institute of Stomatology & Laboratory of Oral Tissue Engineering, School and Hospital of Stomatology, Fujian Medical University, Fuzhou 350002, People’s Republic of China; Fujian Key Laboratory of Oral Diseases & Fujian Provincial Engineering Research Center of Oral Biomaterial & Stomatological Key Laboratory of Fujian College and University, School and Hospital of Stomatology, Fujian Medical University, Fuzhou 350002, People’s Republic of China; Institute of Stomatology & Laboratory of Oral Tissue Engineering, School and Hospital of Stomatology, Fujian Medical University, Fuzhou 350002, People’s Republic of China; Fujian Key Laboratory of Oral Diseases & Fujian Provincial Engineering Research Center of Oral Biomaterial & Stomatological Key Laboratory of Fujian College and University, School and Hospital of Stomatology, Fujian Medical University, Fuzhou 350002, People’s Republic of China; Institute of Stomatology & Laboratory of Oral Tissue Engineering, School and Hospital of Stomatology, Fujian Medical University, Fuzhou 350002, People’s Republic of China

**Keywords:** ionic self-complementary peptides, bone marrow mesenchymal stem cells, osteogenic differentiation, vascularization

## Abstract

Regeneration of oral craniofacial bone defects is a complex process, and reconstruction of large bone defects without the use of exogenous cells or bioactive substances remains a major challenge. Hydrogels are highly hydrophilic polymer networks with the potential to promote bone tissue regeneration. In this study, functional peptide Dentonin was loaded onto self-assembled peptide hydrogels (RAD) to constitute functionally self-assembling peptide RAD/Dentonin hydrogel scaffolds with a view that RAD/Dentonin hydrogel could facilitate vascularized bone regeneration in critical-size calvarial defects. The functionalized peptide RAD/Dentonin forms highly ordered β-sheet supramolecular structures via non-covalent interactions like hydrogen bonding, ultimately assembling into nano-fiber network. RAD/Dentonin hydrogels exhibited desirable porosity and swelling properties, and appropriate biodegradability. RAD/Dentonin hydrogel supported the adhesion, proliferation and three-dimensional migration of bone marrow mesenchymal stem cells (BMSCs) and has the potential to induce differentiation of BMSCs towards osteogenesis through activation of the Wnt/β-catenin pathway. Moreover, RAD/Dentonin hydrogel modulated paracrine secretion of BMSCs and increased the migration, tube formation and angiogenic gene expression of human umbilical vein endothelial cells (HUVECs), which boosted the angiogenic capacity of HUVECs. *In vivo*, RAD/Dentonin hydrogel significantly strengthened vascularized bone formation in rat calvarial defect. Taken together, these results indicated that the functionalized self-assembling peptide RAD/Dentonin hydrogel effectively enhance osteogenic differentiation of BMSCs, indirectly induce angiogenic effects in HUVECs, and facilitate vascularized bone regeneration *in vivo*. Thus, it is a promising bioactive material for oral and maxillofacial regeneration.

## Graphical abstract

**Graphical Abstract:**
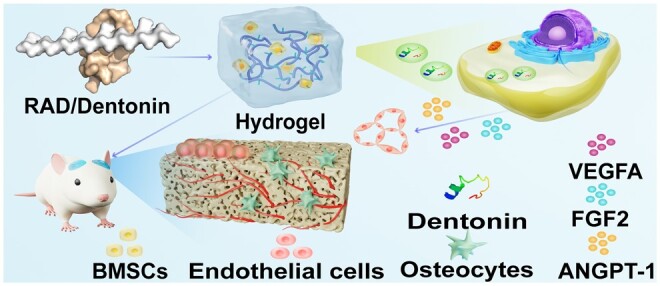

## Introduction

The treatment of large bone defects secondary to trauma, tumors or infection remains a significant challenge for oral and maxillofacial regeneration [1]. Traditionally, autologous and allogeneic transplantation have been used as clinical treatment methods for bone repair. However, several limitations hinder their widespread application, such as limited supply, donor site complications and disease transmission risks [2]. With the development of regenerative medicine, synthetic biomaterial scaffolds have been established to overcome the shortcomings of autologous and allogeneic grafts and enable bone regeneration [3]. Hydrogels, as emerging materials, are highly hydrophilic polymer networks with excellent biocompatibility; they are capable of closely mimicking the extracellular matrix microenvironment and are now widely used in tissue regeneration [4–7].

According to their basic components, hydrogels can be divided into natural polymer-based hydrogels and synthetic polymer-based hydrogels, which showing great potential in biomedicine [8, 9]. The self-assembling peptide RADA16-I (RAD) was synthesized from amino acids and was shown to assembled into nanofiber network hydrogel through non-covalent interactions, exhibiting good biocompatibility and biodegradability [10]. It has also been reported that self-assembled peptide RAD hydrogel has good biocompatibility and provides a favorable microenvironment for periodontal tissue regeneration [11]. However, RAD hydrogels lack bioactivity. To enhance the tissue specificity of RAD, researchers have constructed functionalized peptide hydrogels using peptides with specific biological functions based on different tissue functions [12, 13].

Functional peptides mostly come from the active domains of various proteins already present in human body [14]. Hao *et al*. [15] coupled the biologically active factor parathyroid hormone-related peptide 1 (PTHrP-1) with peptide RAD to develop a multifunctional supramolecular peptide for bone regeneration. The synthetic peptide Dentonin is a functional peptide sequence of extracellular matrix phosphoglycoprotein in humans, containing RGD (integrin-binding motif) and SGDG (glycosaminoglycan attachment motif) sequences. RGD and SGDG can, respectively, bind to integrins and glycosaminoglycans on cell membranes, enhancing cell adhesion and promoting osteogenic activity [16, 17]. Hayashibara *et al*. [18] found that Dentonin can promote the proliferation and differentiation of osteoblasts and can achieve bone regeneration when injected into the skulls of mice. Studies have also shown that combining PDGF-BB with the synthetic peptide Dentonin forms a complex, PDGF-BB/Dentonin, which enhances osteoblast proliferation and osteogenic differentiation [19]. Additionally, Nguyen *et al*. [20] found that Dentonin-modified self-assembling peptide SL-base hydrogels promoted dental pulp stem cells (DPSCs) proliferation and possessed the potential to enhance calcium phosphate deposition. In our previous study, we found that Dentonin bound to the self-assembling peptide RAD to form the functionalized self-assembling peptide RAD/Dentonin, which enhance dentinogenic differentiation of DPSCs [21]. Together, these studies indicate that Dentonin has the potential to promote osteogenic differentiation of cells and is expected to be of use in bone regeneration. However, whether functionalized self-assembling peptide RAD/Dentonin hydrogel could improve bone regeneration *in vivo*, and its underlying mechanisms, remain unclear.

Bone marrow-derived cells possess epigenetic characteristics that allow bone marrow mesenchymal stem cells (BMSCs) to differentiate into osteoblasts, form new mineralized tissue and regenerate large bone defects [22]. Research has shown that functionalized modified hydrogels can regenerate bone by modulating the biological function of BMSCs [23–25]. Therefore, the present study was the first to systematically investigate the effects of RAD/Dentonin hydrogel on cell viability, migration, osteogenic differentiation and pro-angiogenic properties of BMSCs. Furthermore, the efficacy of RAD/Dentonin hydrogel on bone regeneration *in vivo* and its biosafety were evaluated using critical-sized rat calvarial bone defects. This work will not only contribute to our understanding of the bioactivities of RAD/Dentonin hydrogel in bone regeneration but will pave the way for oral and maxillofacial reconstruction using functionalized peptide hydrogels.

## Materials and methods

### Characterization of functionalized self-assembling peptide hydrogel

#### Molecular docking

**Figure 1.**
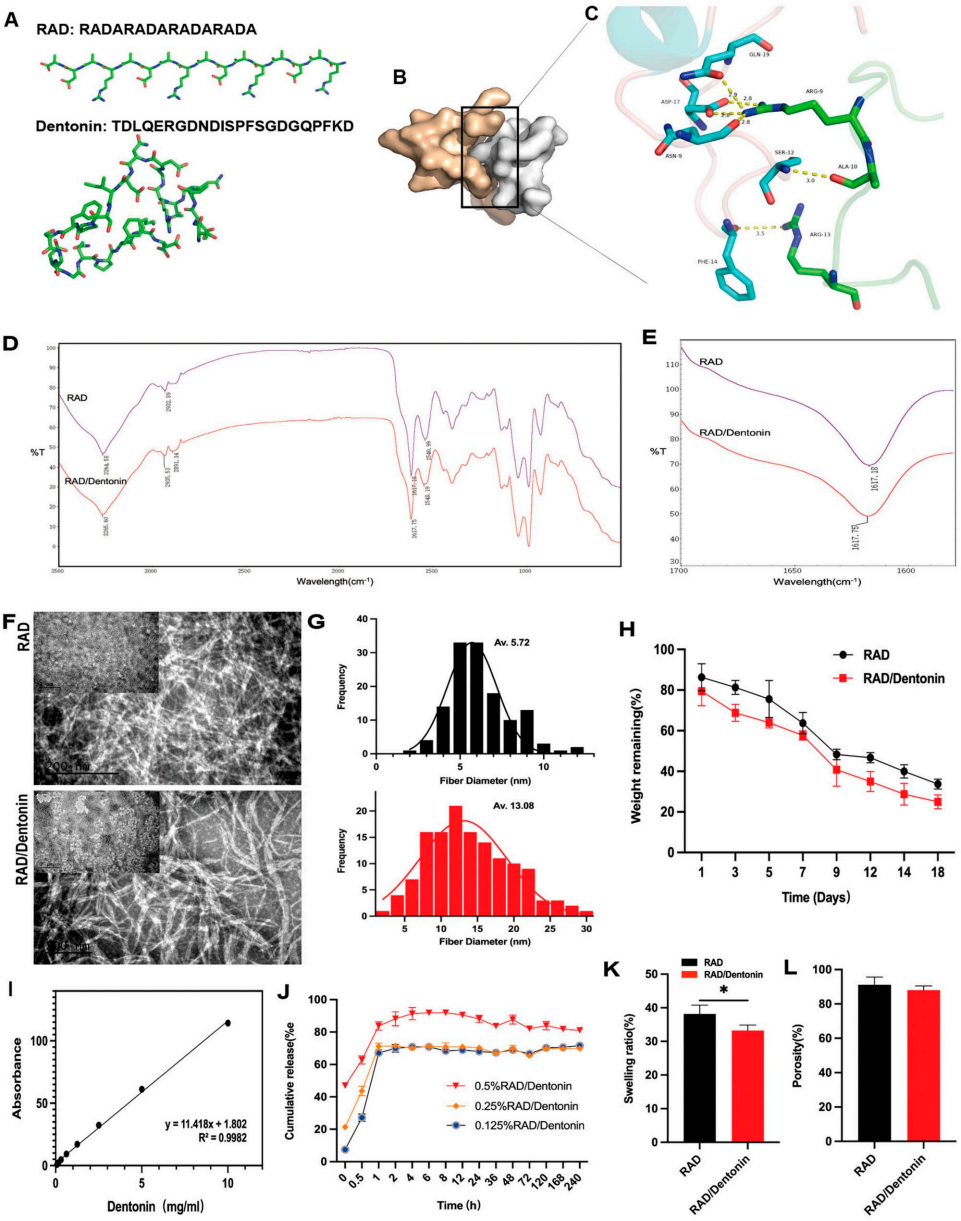
Structure and physicochemical properties of functionalized peptide RAD/Dentonin hydrogel. (**A**) Initial modeling structure of RAD and Dentonin peptides. (**B**) Composite model of RAD and Dentonin ligand peptide. (**C**) Hydrogen bond network of RAD and Dentonin peptide complex; hydrogen bonds depicted as dashed lines. (**D**) FTIR spectra of RAD and RAD/Dentonin hydrogels, showing typical β-sheet structure at ∼1617 cm^-1^ (**E**). (**F**) TEM image of functionalized self-assembling peptide hydrogel (TEM: 200 nm) and (**G**) quantitative analysis of fiber diameter. (**H**) Degradation trends of RAD and RAD/Dentonin hydrogels in PBS. (**I**) Standard curve of Dentonin at 190 nm for different concentrations. (**J**) Cumulative release of Dentonin from RAD/Dentonin hydrogels of different concentrations in PBS. (**K**) Swelling percentage of hydrogels in PBS. (**L**) Porosity (%) of hydrogels.

The RADARADARADARADA and TDLQERGDNDISPFSGDGQPFKD peptide structures were generated using Discovery Studio software, as depicted in Figure 1A. RAD was utilized as the receptor peptide for peptide docking, with Dentonin serving as the ligand structure. Peptide-peptide docking was conducted using the ZDOCK online server (https://zdock.umassmed.edu/). Default parameters were applied, and the conformation with the highest score was selected as the molecular docking conformation. Using the Amber software suite, the molecular mechanical generalized Born surface area (MM/GBSA) method was employed to determine the receptor-ligand binding free energy [26]. The binding free energy calculations were based on snapshots taken at regular intervals from the 60 to 100 ns segment of the molecular dynamics trajectory to ensure adequate sampling and convergence.

#### Fourier transform infrared spectroscopy

A Fourier transform infrared (FTIR) spectrometer (Thermo Scientific Nicolet iS20 instrument, USA) was used to collect spectra in attenuated total reflection mode, detect the transmittance of functionalized peptides, and determine their secondary structures. Spectra were collected with 32 scans at 4 cm^−1^ intervals between wavelengths of 4000 and 400 cm^−1^. The obtained spectra were corrected, smoothed and automatically baseline-corrected using OMNIC software.

#### Transmission electron microscopy

The nanostructures of the functionalized peptides were observed using transmission electron microscopy (TEM) (Tecnai G2 F20, FEI, USA). Load 5 µl of 0.5% (w/v) peptide solution onto a carbon-coated copper grid for 5 min. Then, stain the samples with 5 µl of 2% phosphotungstic acid staining solution for 60 s. The images were acquired using 200 kV accelerating voltage.

#### Degradation and swelling *in vitro*

Peptide hydrogels were prepared based on preliminary experiments [21]. Peptide hydrogels were weighed after formation. Following incubation in PBS solution at 37°C for a specific time point, hydrogel was weighed again. The formula to determines the degradation percentage of each hydrogel was as follows: Degradation = (*W*_1_ − *W*_2_)/*W*_1_ × 100%, where *W*_1_ is the original weight of hydrogel and *W*_2_ is weight of hydrogel after incubation in PBS solution.

Swelling of hydrogels was assessed by submerging the dried hydrogels in PBS at 37°C until saturation. Dried hydrogels were weighed, denoted as *W*_1_, and then submerged in PBS until the hydrogel swelling equilibrated with *W*_2_. Equilibrium swelling was calculated as follows: (*W*_2_ − *W*_1_)/*W*_1_ × 100%.

#### Porosity

Hydrogel porosity was calculated using the liquid immersion method. In brief, hydrogel was freeze-dried and weighed, denoted as *W*_1_. The dried hydrogels were then immersed in anhydrous ethanol until saturated. The excess ethanol was then wiped off with filter paper, and the hydrogels were weighed, denoted as *W*_2_. Hydrogel porosity was calculated as follows: Porosity (%) = (*W*_2_ − *W*_1_)/(*ρ* × *V*) × 100%, where *ρ*  =  0.789 g/ml, which is the density of anhydrous ethanol at 20°C, and *V* is the volume of hydrogel.

#### Release of Dentonin from RAD/Dentonin hydrogel

For the release experiments *in vitro*, different concentrations of RAD/Dentonin hydrogels were immersed in tubes containing PBS. Supernatants were collected at specific time points and replaced with an equal volume of fresh buffer. Measure the concentration of Dentonin in supernatant using UV-Visible spectrophotometer (Thermo Scientific, USA). The cumulative percentage release of Dentonin was calculated from the standard curve.

#### BMSCs isolation and identification

Sprague Dawley (SD) rats were euthanized under ketamine anesthesia by intraperitoneal injection. The intact femurs and tibias were quickly removed under strict aseptic conditions. The ends of bones were trimmed to expose marrow cavity. The bone marrow cavity was repeatedly rinsed with culture medium until the bones turned translucent. Cells were collected and then cultured in Dulbecco’s modified eagle medium (DMEM, Hyclone, USA) supplemented with 10% fetal bovine serum (FBS, Wisent, Canada) and 1% antibiotics. 3–5 generations of cells were available for subsequent experiments.

To assess BMSCs proliferative capacity, cells were stained with crystal violet after 10 days incubation. BMSCs were cultured in DMEM supplemented with 10% FBS, 10 mmol/l β-glycerophosphate, 50 μg/ml L-ascorbic acid phosphate and 10 nmol/l Dexamethasone for osteogenic differentiation. Regarding adipogenic and chondrogenic differentiation, BMSCs were cultured using Adipogenic and Chondrogenic differentiation kits (Cyagen, China). After 4 weeks, BMSCs were stained with Alizarin Red S, Oil Red O and Alcian Blue, respectively. Flow cytometry (BD Accuri C6 Plus, USA) was used to identify BMSCs using antibodies against CD90-FITC, CD45-FITC (BD, USA), CD44-PE and CD31-FITC (Abcam, UK).

#### Cell behavior of BMSCs cultured in functionalized peptide hydrogel

After preparing the functionalized peptide hydrogel, BMSCs were inoculated onto hydrogel and cultured for 1, 4 and 7 days. Then, BMSCs were incubated with Calcein-AM and EthD-1 for 30 min, and images were acquired using an inverted fluorescence microscope (ZEISS, Germany). The number of cells was automatically calculated using ImageJ software to quantitatively analyze live and dead cells. To examine the effect of functionalized peptide hydrogels on BMSCs proliferation, Cell Counting Kit-8 (CCK-8, Dojindo, Japan) was employed on days 1, 3, 5 and 7. After 2 h of incubation, absorbance at 450 nm was measured by a Microplate reader (SpectraMax iD3, USA).

Furthermore, to assess the adhesion of BMSCs on functionalized peptide hydrogel, cells were inoculated onto the hydrogel and cultured for 6, 24 and 72 h. Following this, the cells were fixed with 4% paraformaldehyde and were then permeabilized. Cytoskeleton was stained with Rhodamine-phalloidin (Uelandy, China), the nuclei were stained with DAPI, and acquired images by microscopy. Additionally, migration of BMSCs on the hydrogel was observed using Calcein-AM staining. Confocal laser scanning microscopy (Olympus, Japan) was employed to reconstruct images of BMSCs migration using Z-axis scanning mode with a 10 µm step size.

#### Effect of functionalized peptide hydrogel on BMSCs osteogenesis

BMSCs were cultured on functionalized hydrogel scaffolds for 7 and 14 days, then stained with the BCIP/NBT alkaline phosphatase color development kit (Beyotime, China), and imaged under the microscope. The alkaline phosphatase (ALP) activity of BMSCs was measured according to the ALP activity assay kit (Jiancheng, China) instructions. BMSCs were cultured on hydrogel for 21 days, and calcium deposition in BMSCs was stained using Alizarin Red S solution. Subsequently, the samples were washed with 10% cetylpyridinium chloride for 10 min, and measurements were taken at 570 nm using a Microplate reader. Reverse transcription-quantitative polymerase chain reaction (RT-qPCR) was undertaken to examine the effect of functionalized peptide hydrogel on the differentiation of BMSCs. After 7 and 14 days of inoculation of BMSCs onto hydrogel, cells were lysed using TRIzol reagent. Subsequently, Quantitative reverse transcription kit (Takara, Japan) was employed to extract total RNA from cells. The extracted RNA was reverse-transcribed into cDNA. SYBR Green master mix (Promega, A6001) was applied to analyze target gene expression levels. Primer sequences utilized are listed in Table 1.

**Table 1.** Primer sequences employed for RT-qPCR

Gene	Primers
GAPDH	F: 5′-CGGCAAGTTCAACGGCACAGTCAAGG-3′
	R: 5′-ACGACATACTCAGCACCAGCATCACC-3′
β-catenin	F: 5′-TTACGGCAATCAGGAAAGCAAG-3′
	R: 5′-AGACAGACAGCACCTTCAGC-3′
Runx2	F: 5′-ACTACTCTGCCGAGCTACGA-3′
	R: 5′-AGTGAAACTCTTGCCTCGTCC-3′
ALP	F: 5′-ACAAGGTGGTGGACGGTGAAC-3′
	R: 5′-CGTGAAGCAGGTGAGCCATAGG-3′
BMP-2	F: 5′-AAAGCGTCAAGCCAAACACAAAC-3′
	R: 5′-ACATCACTGAAGTCCACATACAAAGG-3′
OPN	F: 5′-GAGCAGTCCAAGGAGTATAAGC-3′
	R: 5′-AACTCGTGGCTCTGATGTTC-3′
BSP	F: 5′-ACAACACTGCGTATGAAACCTATGAC-3′
	R: 5′-AGTAATAATCCTGACCCTCGTAGCC-3′
Col-1α1	F: 5′-CAACAGACTGGCAACCTCAAGAAG-3′
	R: 5′-CACAAGCGTGCTGTAGGTGAATC-3′
VEGFA	F: 5′-CGTCCTGTGTGCCCCTAAT-3′
	R: 5′-TGGCTTTGGTGAGGTTTGAT-3′
ANGPT-1	F: 5′-TATGGATGTGAATGAAGGAGGATGG-3′
	R: 5′-ACTGCCTCTGACTGGTTATTGC-3′
FGF-2	F: 5′-CTGGCTATGAAGGAAGATGGAC-3′
	R: 5′-CGGTAAGTGTTGTAGTTATTGGAC-3′

After culturing BMSCs on the hydrogel scaffold for 7 days, RIPA lysis buffer (Beyotime, P0013B) was employed to extract cellular proteins. Equal amounts of protein were detached by 10% sodium dodecyl sulfate-polyacrylamide gel electrophoresis. The proteins were transferred onto 0.22 μm PVDF membrane and incubated with the target antibody overnight. Imaging was performed using the ChemiDoc^TM^ XRS+ imaging system. Band intensities were quantified utilizing Image Lab software (BioRad). Immunofluorescence staining was examined for Runx2 (Boster, China) and β-catenin (Servicebio, China) expression in BMSCs cultured on hydrogel, and stained the cytoskeleton with Rhodamine-phalloidin. Images were captured by laser confocal scanning microscopy.

In addition, to investigate the potential mechanism by which the RAD/Dentonin hydrogel affects BMSC osteogenic differentiation, the Wnt pathway antagonist JW74 (MCE, USA) was added to the experiments. RT-qPCR and ALP staining were examined to confirm the effect of RAD/Dentonin hydrogel on the early osteogenic differentiation of BMSCs.

#### Effect of functionalized peptide hydrogel on angiogenesis of HUVECs

The experiment aimed to verify whether the functionalized peptide RAD/Dentonin hydrogel could regulate BMSCs-mediated angiogenesis of HUVECs. After 7 days of seeding BMSCs on hydrogel scaffolds, cell collection was conducted to assess angiogenic gene expression. Primer sequences that were utilized are provided in Table 1. Meanwhile, supernatants from each group were collected, centrifuged and filtered. The collected supernatants were mixed with culture medium at a 1:1 ratio to prepare conditioned medium (CM) [27]. BMSCs were inoculated onto culture plates, and the supernatant was collected and grouped into BMSCs-CM, BMSCs-RAD-CM and BMSCs-RAD/Dentonin-CM, respectively. Additionally, hydrogel extracts were set as controls, namely, RAD-EX and RAD/Dentonin-EX. HUVECs were obtained from the Cell Bank of the Chinese Academy of Sciences and were cultured in cell culture medium supplemented with 10% FBS.

Linear wound scratches were performed to measure the migration capacity of HUVECs. A sterile tip was used to make horizontal scratches approximately 600 μm wide. HUVECs were incubated in each CM group for 12 and 24 h, respectively. The cell migration was calculated according to the following formula: Cell migration rate = ((Initial width − Final width)/Initial width) × 100%. Transwell systems (BD Falcon, USA) with an 8.0 μm pore size were employed to assess the vertical migration of HUVECs. HUVECs were loaded into the upper chamber with 1 × 10^4^ cells per well. CM was added to lower chamber. After 12 h, cells were stained with 0.1% crystal violet for 10 min. To assess the ability of the hydrogel to stimulate angiogenesis, HUVECs were induced to form capillary-like structures by Matrigel (Corning, USA). In brief, HUVECs were mixed with CM from each group and seeded onto the Matrigel. Images were captured using a microscope after 6 h. Tube formation was quantified using ImageJ. To assess the angiogenic capacity of functionalized peptides on HUVECs, RT-qPCR was conducted to investigate angiogenic gene expression in HUVECs. The primer sequences applied are listed in Table 2.

**Table 2.** Primer sequences employed for RT-qPCR

Gene	Primers
GAPDH	F: 5′-ACCCACTCCTCCACCTTTGAC-3′
	R: 5′-TCCACCACCCTGTTGCTGTAG-3′
VEGFA	F: 5′-CGCTTACTCTCACCTGCTTCTG-3′
	R: 5′-TCCAACAATGTGTCTCTTCTCTTCG-3′
eNOS	F: 5′-TTGTCTGCGGCGATGTTACC-3′
	R: 5′-GCGTATGCGGCTTGTCACC-3′
HIF-1α	F: 5′-AGGACACAGATTTAGACTTGGAGATG-3′
	R: 5′-CAGTGGTAGTGGTGGCATTAGC-3′
KDR	F: 5′-CGCAGAGTGAGGAAGGAGGAC-3′
	R: 5′-CCGTAGGATGATGACAAGAAGTAGC-3′
FGFR-1	F: 5′-CTGGGAGAGGGCTGCTTTGG-3′
	R: 5′-CACTTTGGTCACACGGTTGGG-3′
TEK	F: 5′-GCAGAGAACAACATAGGGTCAAGC-3′
	R: 5′-AGGTCATTCCAGCAGAGCCAAG-3′

#### Rat calvarial bone defect

The surgical and animal care procedures were reviewed and approved by the Animal Experiment Supervision Committee of Fujian Medical University (Issue No. IACUC FJMU 2023-0097) and were performed in accordance with the National Institutes of Health Guidelines for Animal Experimentation. The experiments were designed to minimize the number of rats used and every effort was made to diminish pain. SD rats aged 6 weeks, weighing 200–250 g, were chosen for experiment. Rats were anaesthetized by 0.3% pentobarbital intraperitoneally. After routine disinfection, an incision was made along the midline of skull, and the full-thickness skin flap was lifted to expose the skull. A critical size defect of 5 mm diameter was created in the rat skull using a trephine drill. Rats were divided into three groups (*n* = 6) randomly to receive RAD hydrogel, RAD/Dentonin hydrogel and unfilled group, respectively. The incision was closed with interrupted sutures using nylon thread, and the animals were provided with routine postoperative analgesia. The growth status and wound healing of the rats were observed weekly, and their body weight changes were recorded. At 8 weeks post-surgery, euthanasia was performed on the animals, and the skull and viscera were collected for histological analysis.

#### Micro-computed tomography

Skulls were scanned using μCT-100 system (SCANCO Medical AG, Switzerland). The CTAn reconstruction software was employed to reconstruct the raw images of the selected regions. Parameters like percentage of new bone volume (BV/TV), bone mineral density (BMD) and trabecular number (Tb.N) were calculated from quantitative measurements.

#### Histological analysis and immunofluorescence

Following the characterization and cellular functional assessment of the functionalized peptide hydrogels *in vitro*, its toxicity was examined in rat calvarial defect model. Specifically, histological analysis of the heart, liver, spleen, lungs and kidneys was performed. Skulls were decalcified in 20% EDTA for 2 months, followed by dehydration, embedding and sectioning into 4 µm sections. Hematoxylin and eosin (H&E) staining was used to evaluate new bone formation in defect areas. In addition, Mason trichrome and Alizarin Red S staining were performed to identify characteristic cells and mineralized tissue. Immunofluorescence staining was used to assess newly formed vessels and to detect Runx2 (Boster, China), CD31 and Endomucin (Emcn) expression (Servicebio, China). Quantification was performed by Image J software.

### Statistical analysis

All data were statistically analyzed using GraphPad Prism 9 software. Before analysis, the normality and homogeneity of variance of each parameter were checked. Two-sample *t*-tests or one-way analysis of variance (ANOVA) with post-hoc tests were performed for pairwise comparisons. *P *<* *0.05 was considered statistically significant. *P <* *0.05, **P <* *0.01, ***P <* *0.001 and ****P <* *0.0001. Each test was repeated three times.

## Results

### The structure and physicochemical properties of the functionalized peptide RAD/Dentonin hydrogel

Molecular docking revealed that RAD peptide bound within the surface groove of the ligand peptide Dentonin, forming a tight interaction (Figure 1B). Hydrogen bonding occurred between Arg of RAD and Phe, Ser and Asn, while salt bridge interactions potentially involved Gln with Arg and Asp with Arg. The binding free energy between the peptide was −44 kcal/mol, indicating excellent binding affinity (Figure 1C). Further characterization of the stability of functionalized peptide RAD/Dentonin was conducted using FTIR for secondary structure analysis. FTIR spectra exhibited characteristic peptide absorption bands at 3265, 2935, 1617 and 1548 cm^−1^, corresponding to stretching vibrations of -NH_2_, C-H aliphatic and C=O acetyl, and N-H bending vibrations, respectively (Figure 1D). Acetyl stretching vibrations of peptide bonds were observed in the amide I band at 1617 cm^−1^, indicating interactions between peptide secondary structure and β-sheet formation in the 1617 cm^−1^ region (Figure 1E). To analyze the nanostructure of functionalized peptides, TEM was used to observe the hydrogel samples. TEM revealed self-assembly of functionalized peptides into nanofibers, with RAD/Dentonin hydrogel fibers assembling into ‘flattened bands’ with uneven widths and high aspect ratios (Figure 1F). Quantitative analysis of the nanofiber diameters formed by RAD and RAD/Dentonin revealed diameters of 5.72 ± 1.46 nm and 13.08 ± 5.95 nm, respectively (Figure 1G).

RAD and RAD/Dentonin hydrogels exhibited similar degradation characteristics. As shown in Figure 1H, the hydrogel dissolves within the first 3 days, followed by a slow degradation rate. After 18 days of immersion, RAD and RAD/Dentonin hydrogels degraded by approximately 70%. Dentonin exhibited a maximum absorption peak at 190 nm; hence, 190 nm was selected as the detection wavelength. The concentration standard curve formula for Dentonin was *y* = 11.418*x* + 1.802, with *R*^2^ = 0.9982, indicating a linear relationship between Dentonin concentration and OD190 nm values (Figure 1I). Each group initially experienced a burst release effect over 24 h, after which, the release rates stabilized over time. Dentonin release was dependent on the total concentration in the hydrogel. With an increase in concentration, the release of Dentonin also increased. The RAD/Dentonin hydrogel released drugs continuously for at least 48 h (Figure 1J). The liquid immersion method indicated that RAD hydrogel had good porosity (approximately 91.68%), and with the addition of Dentonin, it occupied the internal space of the hydrogel scaffold, resulting in a reduction in the porosity of the RAD/Dentonin hydrogel (approximately 87.44%). The decrease in porosity may be due to the enhanced hydrogen bonding in the hydrogel network. However, there was no significant difference in porosity between RAD and RAD/Dentonin hydrogels (Figure 1K). As shown in Figure 1L, all hydrogels exhibited good swelling properties. Compared to the RAD/Dentonin hydrogel, the single-component RAD hydrogel had a higher swelling rate; swelling rates of RAD and RAD/Dentonin were 38.15% ± 6.7% and 33.22% ± 2.5%, respectively.

### Isolation and identification of BMSCs

BMSCs were isolated from the rat bone marrow and colony formation characteristics, multi-potent differentiation potential and stem cell marker expression were evaluated. Under the microscope, BMSCs exhibited adherent growth (Supplementary Figure S1A), and upon passage, displayed a well-defined cellular morphology (Supplementary Figure S1B). Crystal violet staining was performed to examine the colony growth of BMSCs (Supplementary Figure S1C). Following induction of adipogenesis, osteogenesis and chondrogenesis, BMSCs were able to form mineralized nodules (Supplementary Figure S1D), exhibited high expression of alcian blue-stained acidic polysaccharides (Supplementary Figure S1E), and contained lipid-rich vacuoles (Supplementary Figure S1F), indicative of their multipotential differentiation capacity. Flow cytometry analysis exhibited that BMSCs expressed CD44 and CD90, with a positivity rate greater than or equal to 95%, while surface markers CD45 and CD31 were expressed in less than 2% of the total population (Supplementary Figure S1G).

### Cell compatibility of RAD/Dentonin hydrogel

**Figure 2.**
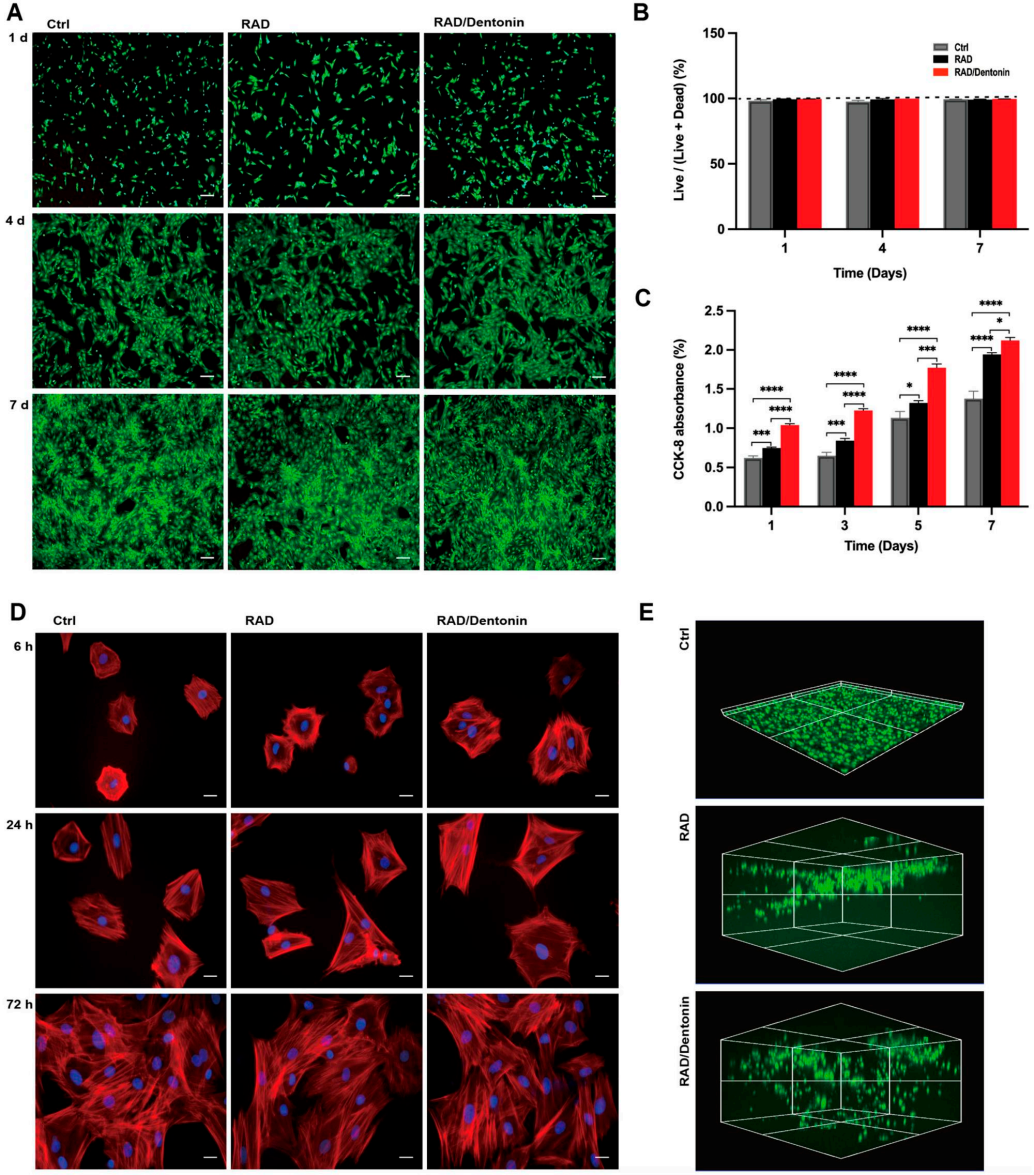
Biocompatibility assessment of functionalized peptide RAD/Dentonin hydrogel scaffold. (**A**) Live/dead double fluorescence staining of BMSCs cultured on hydrogels for 1, 4 and 7 days was conducted, and quantified (**B**); scale bar: 200 µm. (**C**) Proliferation of BMSCs on hydrogel. (**D**) BMSC adhesion on hydrogels at different time points, stained with Phalloidin-DAPI; scale bar: 50 µm. (**E**) BMSCs seeded on tissue culture plates, RAD, and RAD/Dentonin hydrogels, stained with Calcein-AM, were subjected to 3D reconstruction for migration assessment.

Cell cytotoxicity of RAD and RAD/Dentonin hydrogels was evaluated through live/dead fluorescence dual staining. Cells exhibited robust growth on both hydrogel scaffolds, with almost no dead cells observed (Figure 2A). Quantitative analysis (Figure 2B) demonstrated that over 99% of BMSCs stained with green fluorescence were viable on the hydrogel. BMSCs proliferation was measured by CCK-8 assay. RAD exhibited a proliferation-promoting effect on BMSCs compared to controls, and RAD/Dentonin was more proliferative (Figure 2C). Thus, RAD/Dentonin hydrogels demonstrated good cytocompatibility.

Images of BMSCs on the surfaces of the hydrogel indicated excellent cell adhesion. At initial stage of 6 h, BMSCs could successfully attach to the RAD and RAD/Dentonin hydrogel surfaces, and as time progressed, BMSCs on both hydrogel groups exhibited polygonal or elongated spindle-shaped spreading (Figure 2D). Three-dimensional reconstruction of BMSCs migration within the hydrogel was performed using confocal microscopy. Figure 2E presents the reconstructed images of BMSCs migration on RAD, and RAD/Dentonin hydrogels, with BMSCs showing a notably increased migration distance towards the interior of the RAD/Dentonin hydrogel.

### RAD/Dentonin hydrogel promotes osteogenic differentiation of BMSCs *in vitro*

**Figure 3.**
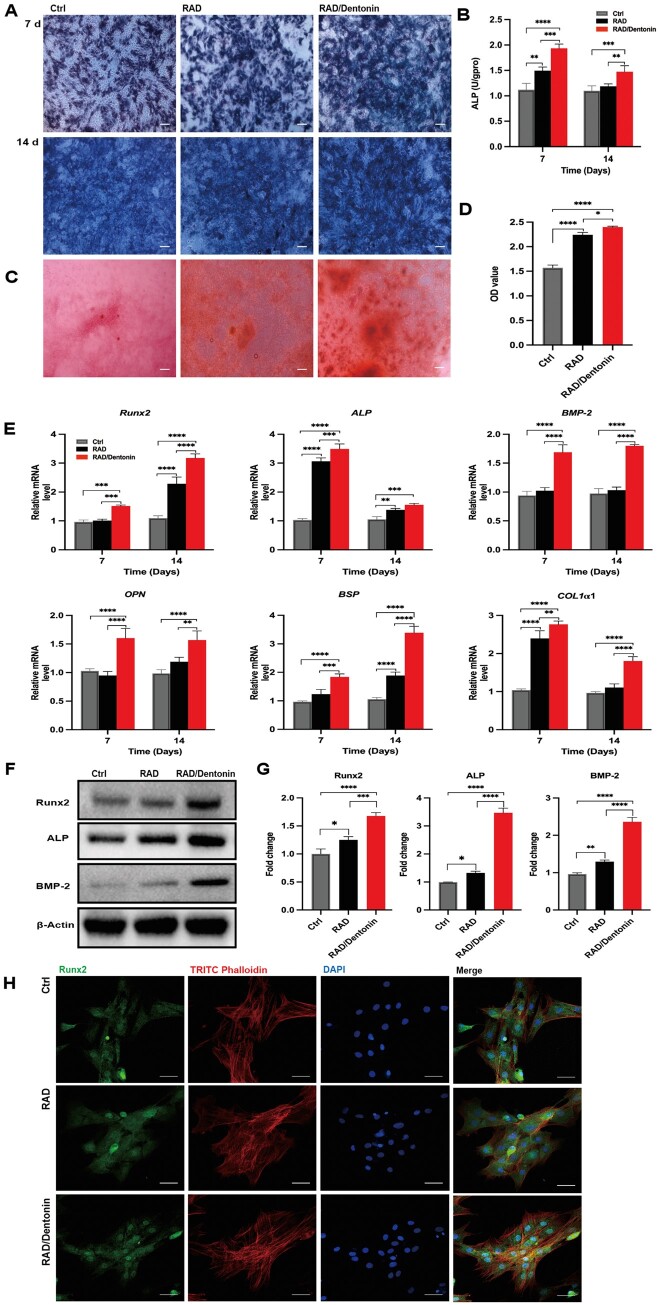
RAD/Dentonin hydrogel promotes osteogenic differentiation of BMSCs. (**A**, **B**) ALP staining and activity assay, and Alizarin Red S staining with quantitative analysis; scale bar: 200 µm (**C**, **D**). (**E**) RT-qPCR to assess osteogenic gene expression in BMSCs. (**F**, **G**) Osteogenic protein expression in BMSCs and quantitative analysis. (**H**) Immunofluorescence staining of Runx2 expression in BMSCs; scale bar: 50 µm.

ALP staining revealed more ALP-positive areas in BMSCs cultured on RAD and RAD/Dentonin hydrogels compared to the control group. Moreover, the staining intensity was notably higher in the RAD/Dentonin group (Figure 3A). ALP activity analysis was consistent with the ALP staining results, indicating that osteogenic differentiation level of BMSCs was significantly increased in RAD/Dentonin hydrogel group (Figure 3B). Additionally, BMSCs cultured on RAD and RAD/Dentonin hydrogel scaffolds exhibited more pronounced Alizarin Red S-positive staining and more calcified nodules compared to controls (Figure 3C). Quantitative analysis of Alizarin red S staining revealed higher mineralization in RAD/Dentonin hydrogels (Figure 3D).

To further assess the impact of RAD/Dentonin hydrogel on the osteogenic differentiation of BMSCs, experiments were designed to assess BMSCs osteogenic gene expression by RT-qPCR. After 7 and 14 days, the expression levels of osteogenic differentiation genes of in BMSCs, including *Runx2*, *ALP*, *BMP-2*, *OPN*, *BSP*, and *COL-1α1*, were elevated on RAD and RAD/Dentonin hydrogels, with more pronounced expression on the RAD/Dentonin hydrogels (Figure 3E). Moreover, the expression levels of the osteogenic proteins Runx2, ALP and BMP-2 were examined (Figure 3F). Osteogenic protein expression was significantly increased in the RAD/Dentonin hydrogel group compared to RAD group, and this was confirmed by quantitative analysis of band density (Figure 3G). Subsequently, immunofluorescence staining was conducted to explore the impact of hydrogel on Runx2 expression during BMSCs differentiation. As displayed in Figure 3H, Runx2 expression was significantly higher in the nuclei of BMSCs in RAD/Dentonin hydrogel group compared to the RAD and control groups.

### RAD/Dentonin hydrogel induces osteogenic differentiation of BMSCs through Wnt/β-catenin signaling pathway

**Figure 4.**
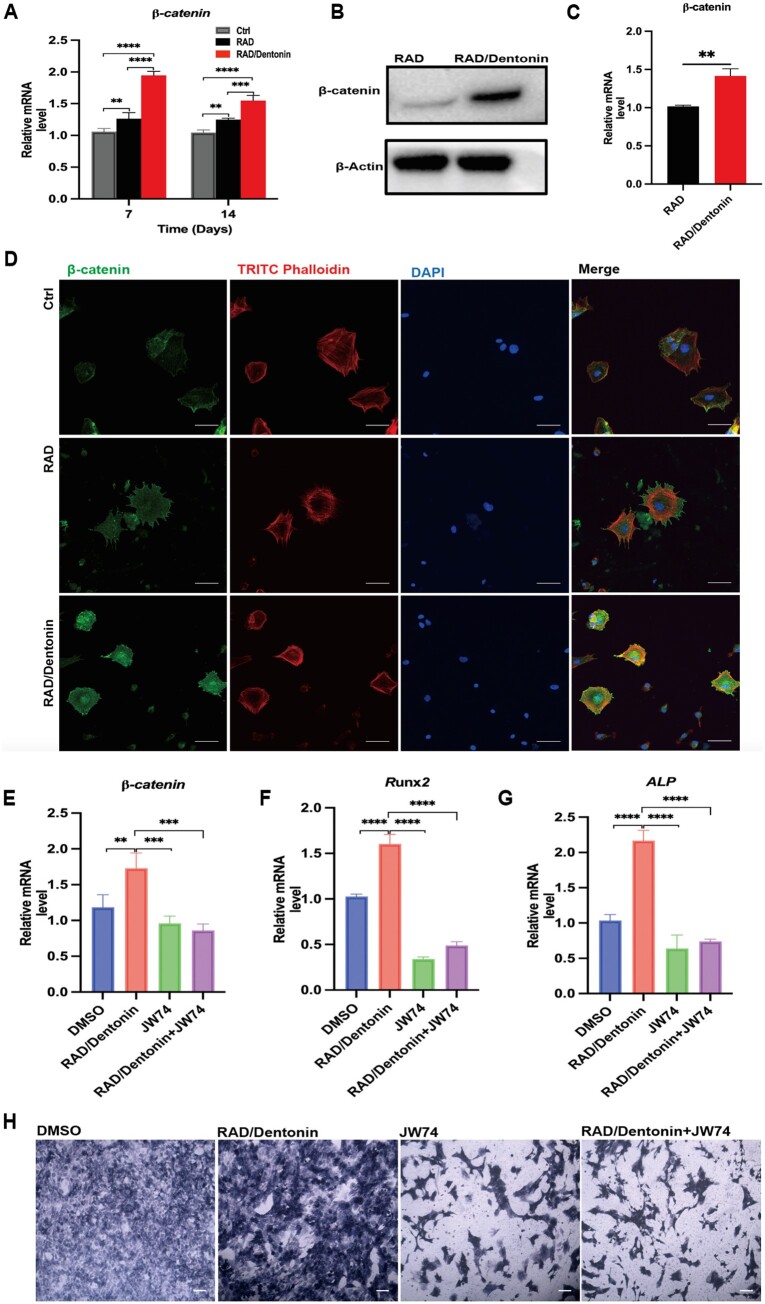
RAD/Dentonin hydrogel regulates BMSCs osteogenic differentiation through Wnt/β-catenin signaling pathway. (**A**) RT-qPCR was conducted to estimate β*-catenin* gene expression in BMSCs. (**B**, **C**) Western blot analysis and quantification of β-catenin protein expression levels in BMSCs. (**D**) Immunofluorescence staining of β-catenin expression in BMSCs cultured on hydrogel; scale bar: 50 µm. (**E**–**G**) RT-qPCR was performed to examine the expression of osteogenic genes in BMSCs treated with the Wnt/β-catenin signaling pathway inhibitor JW74. (**H**) ALP staining of BMSCs treated with JW74; scale bar: 200 µm.

After BMSCs were incubated on RAD/Dentonin hydrogels for 7 and 14 days, RT-qPCR exhibited a marked increase in *β-catenin* expression (Figure 4A). Additionally, β-catenin was assessed at the protein level (Figure 4B). Compared to RAD group, the RAD/Dentonin hydrogel group showed a significant increase in β-catenin protein expression (Figure 4C). Subsequently, immunofluorescence staining (Figure 4D) revealed that RAD/Dentonin hydrogel induced β-catenin translocation to the nucleus. Furthermore, Wnt antagonist JW74 was applied to detect the potential role of RAD/Dentonin hydrogel in inducing osteogenic differentiation of BMSCs. RT-qPCR (Figure 4E–G) and ALP staining (Figure 4H) demonstrated that RAD/Dentonin significantly promoted osteogenic activity of BMSCs. Taken together, these findings indicate that RAD/Dentonin hydrogel might stimulate the osteogenic differentiation of BMSCs through Wnt/β-catenin signaling pathway.

### Indirect regulation of HUVECs angiogenesis by functionalized peptide RAD/Dentonin hydrogel

**Figure 5.**
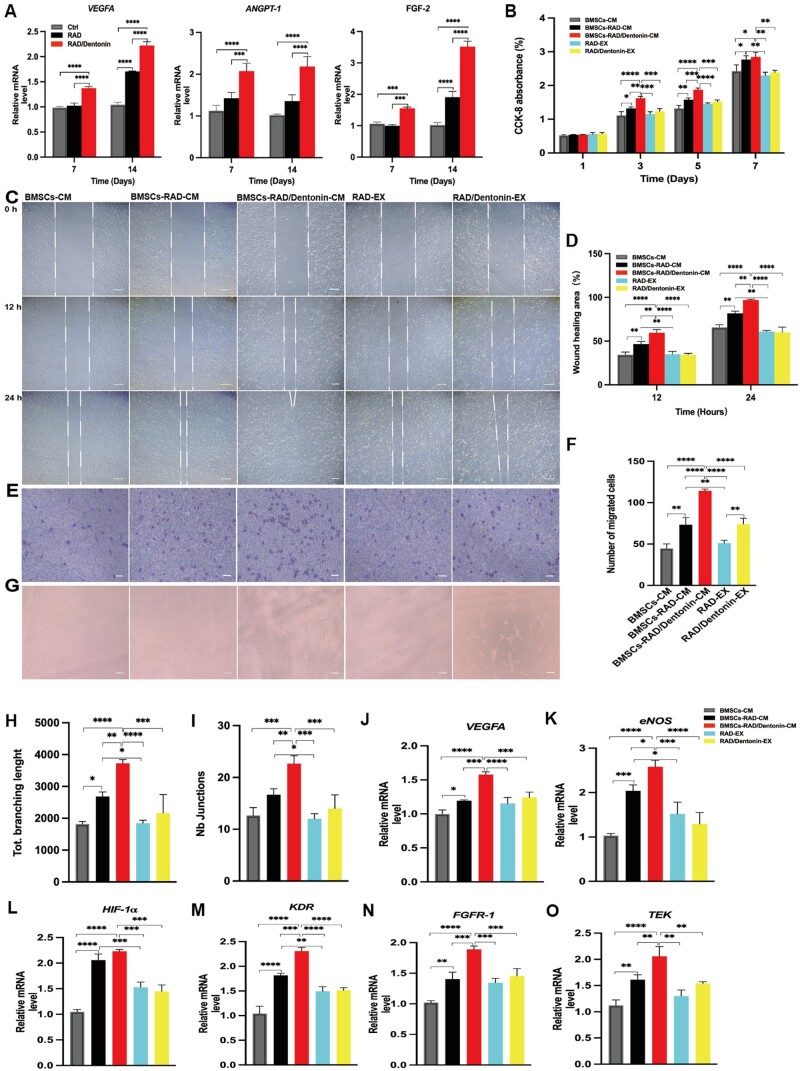
Evaluation of the indirect regulation of angiogenesis in HUVECs by RAD/Dentonin hydrogel. (**A**) Expression of angiogenic genes in BMSCs of each group. (**B**) Proliferation capacity of HUVECs in CM of the different groups. (**C**) Microscopic images of HUVECs migration and quantitative analysis of horizontal migration in different CM groups (**D**). (**E**) Transwell assay to detect effect of different CM groups on vertical migration of HUVECs and quantitative analyses (**F**); scale bar: 200 μm. (**G**) Tube formation in HUVECs induced by different CM groups, and quantitative assessment (**H**, **I**); scale bar: 100 μm. (**J**–**O**) RT-qPCR analysis of the effect of different CM groups on HUVEC gene expression.

Angiogenesis is closely associated with bone formation and remodeling processes. To further understand the role of RAD/Dentonin hydrogel in angiogenesis, angiogenic genes in BMSCs were analyzed. Figure 5A shows that the expression levels of the angiogenic genes *VEGFA, ANGPT-1* and *FGF-2* were increased in BMSCs cultured on RAD and RAD/Dentonin hydrogels as compared with control, and RAD/Dentonin hydrogel demonstrated a higher potential for promoting angiogenesis.

Figure 5B illustrates the cell viability analysis of cells cultured with CM and extract. The cell viability of each sample increased with the culture time. BMSCs-RAD-CM and BMSCs-RAD/Dentonin-CM both promoted cell proliferation, with BMSCs-RAD/Dentonin-CM having a more pronounced effect. Subsequently, the effect of each group of CM on HUVECs migration was investigated. Scratch assays indicated that both BMSCs-RAD-CM and BMSCs-RAD/Dentonin-CM stimulated the migration of HUVECs over time, thereby increasing the rate of scratch closure; the migration rate stimulated by BMSCs-RAD/Dentonin-CM was faster (Figure 5C). The quantitative analysis of scratch assay results was consistent with microscopic observations (Figure 5D). Furthermore, Transwell assays were conducted to measure the influence of CM on vertical migration of HUVECs. After 12 h, RAD-EX and RAD/Dentonin-EX showed no statistically effect on the biological behaviors of HUVECs, whereas BMSCs-RAD-CM and BMSCs-RAD/Dentonin-CM boosted HUVECs migration; BMSCs-RAD/Dentonin-CM exhibited the maximum activation of the paracrine function of BMSCs and significantly induced horizontal and vertical migration of HUVECs (Figure 5E and F).

Furthermore, the angiogenic potential of CM in each group was assessed by tube formation assays. As shown in Figure 5G, BMSCs-RAD-CM and BMSCs-RAD/Dentonin-CM formed a greater number of capillary-like structures compared to RAD-EX and RAD/Dentonin-EX. In addition, HUVECs under BMSCs-RAD/Dentonin-CM stimulation exhibited longer tube lengths and more junctions (Figure 5H and I). Meanwhile, the expression levels of HUVEC genes induced by CM were evaluated in each group, including *VEGFA*, *eNOS*, *HIF-1α*, *KDR*, *FGFR-1* and *TEK* (Figure 5J–O). In contrast to RAD-EX and RAD/Dentonin-EX, BMSCs-RAD-CM and BMSCs-RAD/Dentonin-CM notably enhanced angiogenic gene expression, with BMSCs-RAD/Dentonin-CM showing superior pro-angiogenic capacity *in vitro*.

### Enhanced bone regeneration *in vivo* by functionalized peptide RAD/Dentonin hydrogel

**Figure 6.**
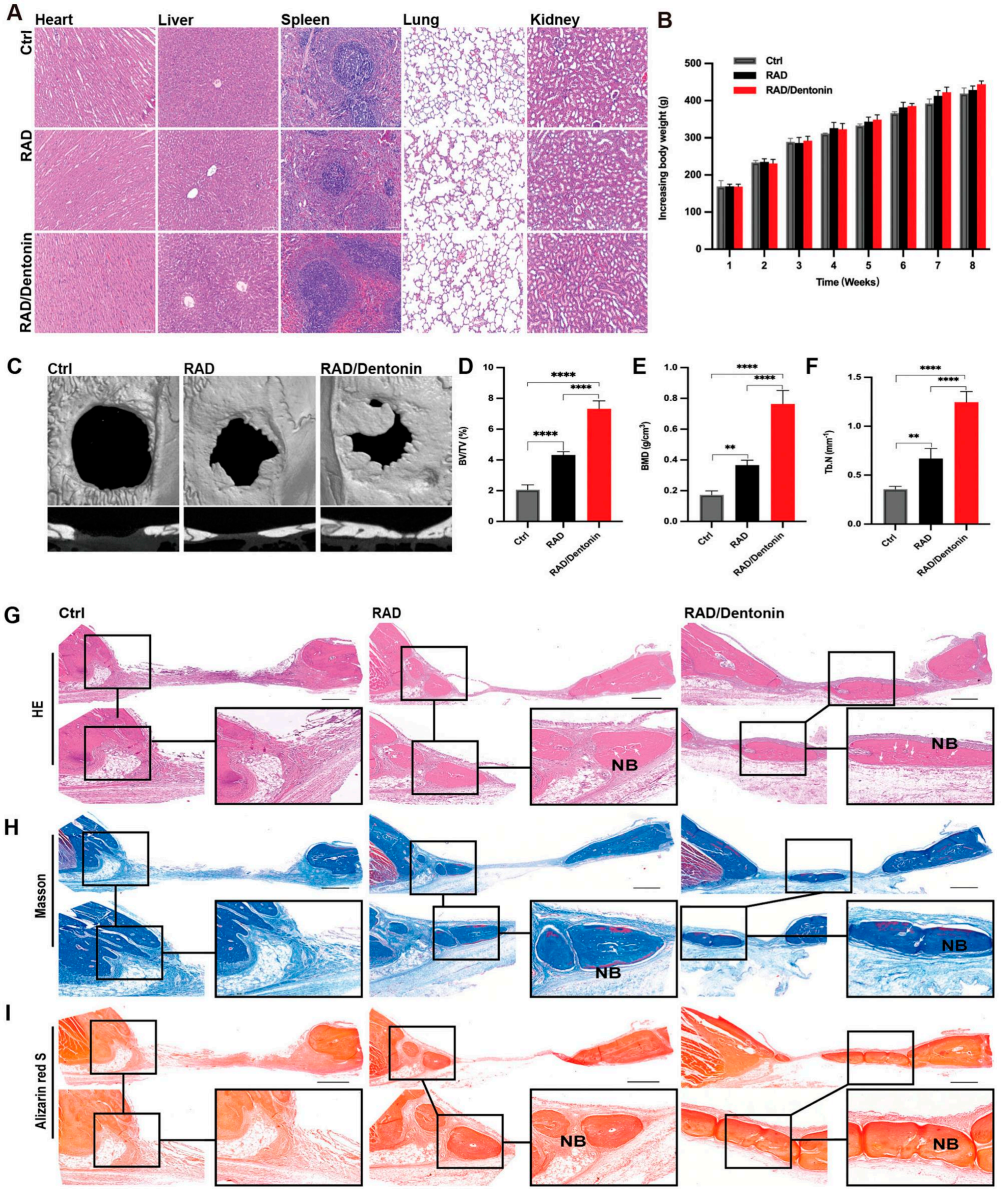
Functionalized RAD/Dentonin hydrogel toxicity and vascularized bone regeneration in rat cranial defects. (**A**) H&E staining of rat viscera with hydrogel implantation 8 weeks later; scale bar: 200 µm. (**B**) Weight change after hydrogel implantation in rats. (**C**) Micro-CT images showing 3D reconstruction and representative coronal sections of cranial defect area, with quantitative analysis of BV/TV, BMD and Tb.N (**D**–**F**). Histological evaluation of rat cranial defect regeneration through (**G**) H&E, (**H**) Masson’s trichrome and (**I**) Alizarin Red S staining. New bone and newly formed vessels are denoted as NB and white arrows, respectively. Low magnification 200 µm, high magnification 100 µm and 50 µm, respectively.

All animals survived the implantation surgery and exhibited good postoperative recovery, with no statistically significant differences in body weight (Figure 6B). H&E staining displayed no abnormalities in histological structures including heart, liver, spleen, lungs and kidneys (Figure 6A). RAD and RAD/Dentonin hydrogel scaffolds demonstrated excellent biocompatibility *in vivo*.

The effect of the functionalized peptide hydrogel on bone regeneration in defect areas was assessed by micro-computed tomography (micro-CT) and histology. Micro-CT images showed relatively little new bone formation in RAD hydrogel-filled areas, with bone thickness decreasing inwardly from the defect edges. However, the RAD/Dentonin hydrogel-filled defects formed a larger area of new bone, connecting the defects with a higher bone thickness (Figure 6C). Micro-CT quantitative analysis revealed that both RAD and RAD/Dentonin hydrogels increased BMD in defect area, but regenerated BMD was higher in RAD/Dentonin hydrogel group. Additionally, BV/TV and Tb. N showed similar trends among the groups (Figure 6D–F). Compared to RAD and control groups, RAD/Dentonin hydrogel evidently improved new bone regeneration in defect area.

H&E staining further confirmed the trends observed in micro-CT. Figure 6G displays that control group had fibrous connective tissue composed of fibroblasts in central area of defect. In contrast, RAD group exhibited new bone tissue and collagen fibers at the edges of the defect. Moreover, RAD/Dentonin hydrogel induced a larger volume of new bone tissue, with the defect almost completely healed after 8 weeks of treatment, and newly formed vessels were visible within the newly formed bone tissue. Additionally, Masson’s trichrome staining was performed on the regenerated area (Figure 6H). The RAD group exhibited smaller bone island formation at the defect edges, increased mineralization of collagen fibers near the defect end, and light-blue staining of collagen was observed at the center of the defects. Moreover, the defect filled with RAD/Dentonin hydrogel gradually formed lamellar structure of mineralized matrix, and osteocytes and new blood vessels enveloped within these bone islands verify the vitality of the newly formed bone. Consistent with Masson’s trichrome staining, Alizarin Red S (Figure 6I) showed that the maximum area of calcification in RAD/Dentonin hydrogel group, followed by RAD hydrogel. This highlights the capacity of RAD/Dentonin hydrogel to guide mineralization of regenerated bone.

### RAD/Dentonin hydrogel induces osteogenesis by promoting H-type angiogenesis

**Figure 7.**
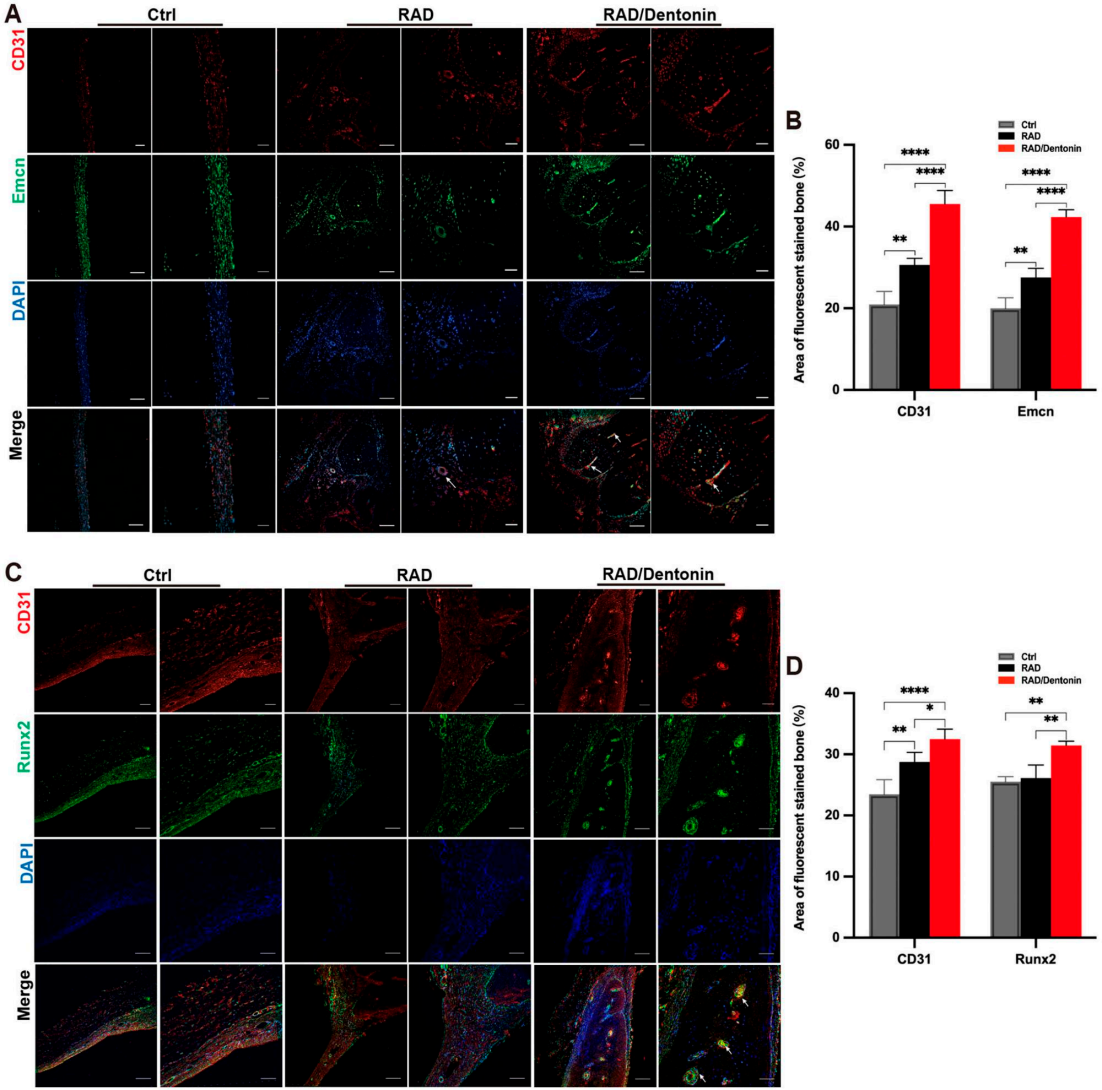
Effect of functionalized RAD/Dentonin hydrogel on angiogenesis-osteogenesis coupling in rat calvarial defects. Immunofluorescence staining images of CD31 and Emcn (A), Runx2 and CD31(**C**), and quantitative analysis of fluorescence intensity (**B**, **D**). Positive cells are indicated by white arrows. Scale bars: 200 µm, 100 µm.

Immunofluorescence staining for Emcn, CD31 and Runx2 expression was performed on calvarial defects filled for 8 weeks. Immunofluorescence staining (Figure 7A) showed that RAD/Dentonin hydrogel scaffold induced more CD31 and Emcn high-positive cells. Fluorescence quantification displayed a stronger effect of RAD/Dentonin (Figure 7B). Additionally, immunostaining revealed that Runx2-positive osteoblasts were selectively distributed around the H-type endothelial cells (Figure 7C), with the RAD/Dentonin hydrogel group showing a higher expression of Runx2-positive osteoblasts (Figure 7D).

## Discussion

Throughout bone development and healing, osteogenesis and angiogenesis are tightly intertwined [28]. Therefore, tissue-engineered scaffolds should provide osteoconductive structures and should also deliver bioactive factors to guide desirable bone regeneration [29]. In this study, RAD/Dentonin hydrogel accelerated vascularized bone regeneration in defect areas. The findings exhibited that RAD/Dentonin hydrogel enhanced BMSCs adhesion, proliferation and three-dimensional migration, and also regulated BMSCs osteogenic differentiation by Wnt/β-catenin signaling pathway. Additionally, BMSCs cultured on RAD/Dentonin hydrogel exhibited enhanced angiogenesis potential through the secretion of angiogenic cytokines. Micro-CT reconstruction and histological results of rat calvarial defects indicated that RAD/Dentonin hydrogel promoted vascularized bone regeneration *in vivo.* These results offer new angles for the application of functionalized self-assembling peptide RAD/Dentonin hydrogels in oral and maxillofacial bone reconstruction.

Preliminary experiments using circular dichroism spectroscopy revealed that functionalized peptide RAD/Dentonin is rich in β-sheet structures. In our previous study, scanning electron microscope showed that the RAD and RAD/Dentonin peptides can self-assemble and cross-link to form a network structure [21]. In this study, molecular docking studies indicated that RAD and Dentonin have excellent binding affinity and can form stable complexes. Moreover, FTIR spectroscopy further confirmed β-sheet structure formation. TEM revealed that RAD/Dentonin hydrogel formed a nano-fibrous network structure, and the addition of Dentonin increased the diameter of the fibers. The findings are similar to the structures of other functionalized peptides related to RAD hydrogel construction [30]. The porosity, swelling and degradation behavior of hydrogel scaffolds are crucial for cells to fulfil their biological functions during tissue regeneration [31]. The current results suggest that the addition of Dentonin led to a reduction in porosity, swelling and degradation behavior of hydrogel, but no statistically significant differences were observed. Moreover, the degradation and release patterns of the RAD/Dentonin hydrogel in the current study were relatively stable, consistent with the findings reported by Zhang *et al.* [29]. Although hydrogel degrade rapidly in the early stage, approximately 20% of the hydrogel remains after 18 days of degradation experiments. One study reported that the rapid degradation of hydrogels may reduce osteoclast activity and inflammatory infiltration, potentially leading to more complete alveolar bone healing [32]. We presume that the rapid degradation in the early stage of the RAD/Dentonin hydrogel contributes to its biological function.

Materials for bone regeneration should have the ability to enrich BMSCs into the defect area and then guide osteogenesis and angiogenesis [33]. The current study demonstrates that RAD and functionalized peptide RAD/Dentonin hydrogels had no adverse effects on BMSCs. Furthermore, RAD/Dentonin hydrogel exhibited good cellular compatibility and promoted the migration and proliferation of BMSCs into the hydrogel interior. These maybe the RGD motif in Dentonin has a high affinity for integrin receptors and that the functionalized RGD peptide enhances the adhesion of nanoscale materials to cells expressing these receptors. The presentation pattern of the RGD motif in peptide hydrogel can significantly affect the cell attachment, extension and migration [16, 34]. These results are similar to our previous finding that DPSCs were found to have better proliferative activity in RAD/Dentonin hydrogel [21].

Osteogenesis plays a vital role in oral-maxillofacial bone defects. To strengthen the osteogenic capacity of BMSCs, various bone regeneration strategies have been adopted [35–38]. In present study, *in vitro* bio-mineralization activity assays, ALP activity and Alizarin Red S staining results all indicated that the three-dimensional structure of RAD and RAD/Dentonin hydrogels could maintain BMSCs’ biological functions. In addition, the functionalized peptide RAD/Dentonin hydrogel significantly up-regulated osteogenic genes *Runx2*, *ALP*, *BMP-2, OPN*, *BSP* and *COL-1α1* in BMSCs, which significantly contributed to the osteogenic differentiation of BMSCs. *Runx2*, an early marker of osteogenesis, can effectively enhance the osteogenic potential of BMSCs when activated [39]. *ALP* is one of the earliest genes involved in mineralization of hard tissues [40]. *COL-I* can stimulate osteoblast adhesion and differentiation, and it is fundamental to bone formation [41]. *BSP* is abundantly expressed in mineralized tissues and directly promotes the production of mineralized matrix [42]. *OPN* has multifunctional roles in regulating mineralization reactions and cell activity [43]. These results collectively indicate that RAD/Dentonin hydrogel promotes osteogenesis of BMSCs. We speculated that Dentonin is released by degradation from RAD/Dentonin hydrogel and taken up by cells. Similar results have been found in previous studies of Dentonin-containing functionalized peptides [21].

Wnt/β-catenin signaling pathway forms a critical regulator of osteogenic differentiation in BMSCs [44, 45]. To further elucidate mechanism by which RAD/Dentonin hydrogel stimulates osteogenesis in BMSCs, experiments were performed to verify the regulation of the Wnt/β-catenin signaling pathway during co-culture of BMSCs with RAD/Dentonin hydrogel. RT-qPCR and Western blot results demonstrated that BMSCs treated with RAD/Dentonin hydrogel showed increased β-catenin expression and activated of Wnt/β-catenin signaling pathway. JW74 has been shown to antagonize classical Wnt/β-catenin signaling [46]. To further validate this mechanism, the Wnt signaling of cells was inhibited via JW74 and subsequently, the expression levels of *β-catenin*, *Runx2* and *ALP* were inhibited, and ALP activity was reduced in BMSCs cultured on RAD/Dentonin hydrogels. To the best of our knowledge, this study is the first to describe the signaling pathways through which RAD/Dentonin hydrogels affect the osteogenic differentiation of BMSCs.

Vascular formation is essential for bone regeneration [47, 48]. Our findings indicated RAD and RAD/Dentonin hydrogels maintained BMSCs’ biological functions and increased angiogenic genes *VEGFA*, *ANGPT-1* and *FGF-2* expression, with a more pronounced facilitating effect of RAD/Dentonin hydrogel. Compared to RAD-EX and RAD/Dentonin-EX, BMSCs-RAD-CM and BMSCs-RAD/Dentonin-CM showed good regulation of proliferation, migration and functional tube formation in HUVECs. The expression levels of the angiogenic genes *VEGFA*, *eNOS*, *HIF-1α*, *KDR*, *FGFR-1* and *TEK* were significantly increased in HUVECs stimulated by BMSCs-RAD/Dentonin-CM. BMSCs, as the fundamental cells in bone tissue formation, can differentiate into osteoblasts as well as paracrine cytokines that adjust blood vessel formation process [49, 50]. Studies have shown that low-temperature deposition modeling of sponge-like scaffolds effectively improves the paracrine function of mesenchymal stem cells (MSCs), increasing *VEGFA*, *eNOS* and *ANGPT-1* gene expression, improving endothelial cell function and promoting vascular formation [33]. Our previous studies revealed that BMSCs can regulate endothelial cell vascularization through paracrine effects [51]. The biomimetic structure of the scaffold forms a microenvironment that enhances cell-to-cell and cell-matrix interactions, thereby augmenting the paracrine effects of MSCs [52, 53]. Additionally, studies have shown that MSCs in RGD-modified hyaluronic acid hydrogels secrete more VEGF compared to those in unmodified controls [54, 55]. Thus, we propose that the enhanced paracrine activity of BMSCs co-cultured with the RAD/Dentonin hydrogel is attributed to the nanofiber structure of the hydrogel and the presence of Dentonin containing the RGD motif. However, the underlying mechanisms need to be further investigated.

To date, rat calvarial bone defects have been adopted in many studies to evaluate the properties of biomaterials for bone regeneration [23, 56, 57]. In the current study, the implantation process of functionalized self-assembling peptide hydrogels into rat calvarial defects was completed without any residual gel, verifying the biodegradability of the hydrogel and its integration with surrounding tissues. Moreover, based on body weight measurements, overall health condition of rats across all groups remained consistent. Histopathological examination of important organs revealed no adverse changes, highlighting the *in vivo* biocompatibility of the hydrogels. These findings align with earlier studies on the toxicological safety of functionalized self-assembling peptide hydrogels [58].

Furthermore, bone regeneration was more evident with RAD/Dentonin hydrogel in the cranial defect model. Micro-CT reconstruction revealed that new bone in RAD/Dentonin hydrogel group had almost filled the defect area. Histological staining results demonstrated the presence of new vessels within regenerated bone, indicating successful vascularization; this is crucial for maintaining bone tissue. CD31 is an important marker of endothelial cell migration and blood vessel formation [59]. Emcn is an endothelium-specific salivary hormone that is specifically expressed by the endothelium of veins and capillaries [60]. Studies have indicated that endothelial cells highly expressing the markers CD31 and Emcn, referred to as type H vessels, supply niche signals to perivascular cells for bone tissue regeneration [61]. Qiu *et al*. [62] reported that a porous polycaprolactone/hydroxyapatite—dimethoxyamine using 3D printing can significantly boost H-type vascularization in bone tissue with high CD31 and Emcn expression. In the present study, the formation of vascularized bone tissue in the defect area was evaluated using immunofluorescence staining. The results showed that both RAD and RAD/Dentonin hydrogels increased CD31 and EMCN expression, and Runx2 was located near the CD31^hi^/Emcn^hi^ blood vessels; however, RAD/Dentonin induced more CD31 and Emcn high-positive cells. Our finding revealed that RAD/Dentonin hydrogel contribute to the formation of new vessels in bone tissue. However, further exploration of the mechanism by which RAD/Dentonin hydrogel affects H-vessel formation is warranted.

## Conclusions

Overall, this research provides insight into the molecular interactions and stability of functionalized self-assembling peptide RAD/Dentonin hydrogel. This hydrogel exhibited good cellular compatibility, facilitating the migration and proliferation of BMSCs. Moreover, it exhibited the potential to induce differentiation of BMSCs toward osteogenesis through activation of the Wnt/β-catenin pathway. Additionally, RAD/Dentonin hydrogel regulated BMSCs paracrine secretion, promoting the migration and tube formation of HUVECs. The rat calvarial defect model demonstrated the excellent biocompatibility of RAD/Dentonin hydrogel *in vivo*, enhancing the coupling of osteogenesis and angiogenesis in the defect area, thus promoting vascularized bone regeneration. Our results indicate that functionalized peptide RAD/Dentonin hydrogels holds promise as a candidate material for oral and maxillofacial regeneration. Although the RAD/Dentonin hydrogel promoted rat calvarial bone regeneration, improve the degradation properties of the hydrogel to extend the effect of Dentonin are still needed to explore before clinical practice.

## Supplementary Material

rbae106_Supplementary_Data

## References

[1] Awad K , Ahuja N, Yacoub AS, Brotto L, Young S, Mikos A, Aswath P, Varanasi V. Revolutionizing bone regeneration: advanced biomaterials for healing compromised bone defects. Front Aging 2023;4:1217054. 37520216 10.3389/fragi.2023.1217054 PMC10376722

[2] Dewey MJ , Harley BAC. Biomaterial design strategies to address obstacles in craniomaxillofacial bone repair. RSC Adv 2021;11:17809–27. 34540206 10.1039/d1ra02557k PMC8443006

[3] Patel DK , Jung E, Priya S, Won SY, Han SS. Recent advances in biopolymer-based hydrogels and their potential biomedical applications. Carbohydr Polym 2024;323:121408. 37940291 10.1016/j.carbpol.2023.121408

[4] Liu S , Wang YN, Ma B, Shao J, Liu H, Ge S. Gingipain-responsive thermosensitive hydrogel loaded with SDF-1 facilitates in situ periodontal tissue regeneration. ACS Appl Mater Interfaces 2021;13:36880–93. 34324286 10.1021/acsami.1c08855

[5] Chen S , Wang H, Liu D, Bai J, Haugen HJ, Li B, Yan H. Early osteoimmunomodulation by mucin hydrogels augments the healing and revascularization of rat critical-size calvarial bone defects. Bioact Mater 2023;25:176–88. 36817825 10.1016/j.bioactmat.2023.01.022 PMC9932297

[6] Amirthalingam S , Rajendran AK, Moon YG, Hwang NS. Stimuli-responsive dynamic hydrogels: design, properties and tissue engineering applications. Mater Horiz 2023;10:3325–50. 37387121 10.1039/d3mh00399j

[7] Liu M , Jiang S, Witman N, Wang H, Wang W, Fu W, You Z. Intrinsically cryopreservable, bacteriostatic, durable glycerohydrogel inks for 3D bioprinting. Matter 2023;6:983–99.

[8] Cao H , Duan L, Zhang Y, Cao J, Zhang K. Current hydrogel advances in physicochemical and biological response-driven biomedical application diversity. Signal Transduct Target Ther 2021;6:426. 34916490 10.1038/s41392-021-00830-x PMC8674418

[9] Huang M , Huang Y, Liu H, Tang Z, Chen Y, Huang Z, Xu S, Du J, Jia B. Hydrogels for the treatment of oral and maxillofacial diseases: current research, challenges, and future directions. Biomater Sci 2022;10:6413–46. 36069391 10.1039/d2bm01036d

[10] Zhang S. Self-assembling peptides: from a discovery in a yeast protein to diverse uses and beyond. Protein Sci 2020;29:2281–303. 32939884 10.1002/pro.3951 PMC7586918

[11] Takeuchi T , Bizenjima T, Ishii Y, Imamura K, Suzuki E, Seshima F, Saito A. Enhanced healing of surgical periodontal defects in rats following application of a self-assembling peptide nanofibre hydrogel. J Clin Periodontol 2016;43:279–88. 26788695 10.1111/jcpe.12515

[12] Wang Y , Zhang W, Gong C, Liu B, Li Y, Wang L, Su Z, Wei G. Recent advances in the fabrication, functionalization, and bioapplications of peptide hydrogels. Soft Matter 2020;16:10029–45. 32696801 10.1039/d0sm00966k

[13] Wang R , Wang Z, Guo Y, Li H, Chen Z. Design of a RADA16-based self-assembling peptide nanofiber scaffold for biomedical applications. J Biomater Sci Polym Ed 2019;30:713–36. 31018781 10.1080/09205063.2019.1605868

[14] Morwood AJ , El-Karim IA, Clarke SA, Lundy FT. The role of extracellular matrix (ECM) adhesion motifs in functionalised hydrogels. Molecules 2023;28:4616. 37375171 10.3390/molecules28124616 PMC10302969

[15] Hao Z , Feng Q, Wang Y, Wang Y, Li H, Hu Y, Chen T, Wang J, Chen R, Lv X, Yang Z, Chen J, Guo X, Li J. A parathyroid hormone related supramolecular peptide for multi-functionalized osteoregeneration. Bioact Mater 2024;34:181–203. 38235308 10.1016/j.bioactmat.2023.12.014 PMC10792172

[16] Kumar VB , Tiwari OS, Finkelstein-Zuta G, Rencus-Lazar S, Gazit E. Design of functional RGD peptide-based biomaterials for tissue engineering. Pharmaceutics 2023;15:345. 36839667 10.3390/pharmaceutics15020345 PMC9967156

[17] Liu H , Li W, Gao C, Kumagai Y, Blacher RW, DenBesten PK. Dentonin, a fragment of MEPE, enhanced dental pulp stem cell proliferation. J Dent Res 2004;83:496–9. 15153459 10.1177/154405910408300612

[18] Hayashibara T , Hiraga T, Yi B, Nomizu M, Kumagai Y, Nishimura R, Yoneda T. A synthetic peptide fragment of human MEPE stimulates new bone formation in vitro and in vivo. J Bone Miner Res 2004;19:455–62. 15040834 10.1359/JBMR.0301263

[19] Vordemvenne T , Paletta JR, Hartensuer R, Pap T, Raschke MJ, Ochman S. Cooperative effects in differentiation and proliferation between PDGF-BB and matrix derived synthetic peptides in human osteoblasts. BMC Musculoskelet Disord 2011;12:263. 22104124 10.1186/1471-2474-12-263 PMC3231994

[20] Nguyen PK , Gao W, Patel SD, Siddiqui Z, Weiner S, Shimizu E, Sarkar B, Kumar VA. Self-assembly of a dentinogenic peptide hydrogel. ACS Omega 2018;3:5980–7. 30023936 10.1021/acsomega.8b00347 PMC6045409

[21] Liu Y , Fan L, Lin X, Zou L, Li Y, Ge X, Fu W, Zhang Z, Xiao K, Lv H. Functionalized self-assembled peptide RAD/Dentonin hydrogel scaffold promotes dental pulp regeneration. Biomed Mater 2021;17:015009. 10.1088/1748-605X/ac3928 34768244

[22] Hochmann S , Ou K, Poupardin R, Mittermeir M, Textor M, Ali S, Wolf M, Ellinghaus A, Jacobi D, Elmiger JAJ, Donsante S, Riminucci M, Schäfer R, Kornak U, Klein O, Schallmoser K, Schmidt-Bleek K, Duda GN, Polansky JK, Geissler S, Strunk D. The enhancer landscape predetermines the skeletal regeneration capacity of stromal cells. Sci Transl Med 2023;15:eabm7477. 36947595 10.1126/scitranslmed.abm7477

[23] Liu Y , Zhu Z, Pei X, Zhang X, Cheng X, Hu S, Gao X, Wang J, Chen J, Wan Q. ZIF-8-modified multifunctional bone-adhesive hydrogels promoting angiogenesis and osteogenesis for bone regeneration. ACS Appl Mater Interfaces 2020;12:36978–95. 32814397 10.1021/acsami.0c12090

[24] Li R , Zhou C, Chen J, Luo H, Li R, Chen D, Zou X, Wang W. Synergistic osteogenic and angiogenic effects of KP and QK peptides incorporated with an injectable and self-healing hydrogel for efficient bone regeneration. Bioact Mater 2022;18:267–83. 35387156 10.1016/j.bioactmat.2022.02.011 PMC8961307

[25] Huang C , Ye Q, Dong J, Li L, Wang M, Zhang Y, Zhang Y, Wang X, Wang P, Jiang Q. Biofabrication of natural Au/bacterial cellulose hydrogel for bone tissue regeneration via in-situ fermentation. Smart Materials in Medicine 2023;4:1–14.

[26] Kollman PA , Massova I, Reyes C, Kuhn B, Huo S, Chong L, Lee M, Lee T, Duan Y, Wang W, Donini O, Cieplak P, Srinivasan J, Case DA, Cheatham TE. 3rd. Calculating structures and free energies of complex molecules: combining molecular mechanics and continuum models. Acc Chem Res 2000;33:889–97. 11123888 10.1021/ar000033j

[27] Novello S , Tricot-Doleux S, Novella A, Pellen-Mussi P, Jeanne S. Influence of periodontal ligament stem cell-derived conditioned medium on osteoblasts. Pharmaceutics 2022;14:729. 35456563 10.3390/pharmaceutics14040729 PMC9028528

[28] Kusumbe AP , Ramasamy SK, Adams RH. Coupling of angiogenesis and osteogenesis by a specific vessel subtype in bone. Nature 2014;507:323–8. 24646994 10.1038/nature13145 PMC4943525

[29] Zhang R , Liu Y, Qi Y, Zhao Y, Nie G, Wang X, Zheng S. Self-assembled peptide hydrogel scaffolds with VEGF and BMP-2 enhanced in vitro angiogenesis and osteogenesis. Oral Dis 2022;28:723–33. 33512051 10.1111/odi.13785

[30] Tao H , Wu Y, Li H, Wang C, Zhang Y, Li C, Wen T, Wang X, He Q, Wang D, Ruan D. BMP7-based functionalized self-assembling peptides for nucleus pulposus tissue engineering. ACS Appl Mater Interfaces 2015;7:17076–87. 26197234 10.1021/acsami.5b03605

[31] Kumar A , Won SY, Sood A, Choi SY, Singhmar R, Bhaskar R, Kumar V, Zo SM, Han SS. Triple-networked hybrid hydrogels reinforced with montmorillonite clay and graphene nanoplatelets for soft and hard tissue regeneration. Int J Mol Sci 2022;23:14158. 36430637 10.3390/ijms232214158 PMC9698198

[32] Wang Z , Zhang Y, Yin Y, Liu J, Li P, Zhao Y, Bai D, Zhao H, Han X, Chen Q. High-strength and injectable supramolecular hydrogel self-assembled by monomeric nucleoside for tooth-extraction wound healing. Adv Mater 2022;34:e2108300. 35066934 10.1002/adma.202108300

[33] Lian M , Sun B, Han Y, Yu B, Xin W, Xu R, Ni B, Jiang W, Hao Y, Zhang X, Shen Y, Qiao Z, Dai K. A low-temperature-printed hierarchical porous sponge-like scaffold that promotes cell-material interaction and modulates paracrine activity of MSCs for vascularized bone regeneration. Biomaterials 2021;274:120841. 33984633 10.1016/j.biomaterials.2021.120841

[34] Hersel U , Dahmen C, Kessler H. RGD modified polymers: biomaterials for stimulated cell adhesion and beyond. Biomaterials 2003;24:4385–415. 12922151 10.1016/s0142-9612(03)00343-0

[35] Lu G , Xu Y, Liu Q, Chen M, Sun H, Wang P, Li X, Wang Y, Li X, Hui X, Luo E, Liu J, Jiang Q, Liang J, Fan Y, Sun Y, Zhang X. An instantly fixable and self-adaptive scaffold for skull regeneration by autologous stem cell recruitment and angiogenesis. Nat Commun 2022;13:2499. 35523800 10.1038/s41467-022-30243-5 PMC9076642

[36] Chen M , Zhang Y, Zhang W, Li J. Polyhedral oligomeric silsesquioxane-incorporated gelatin hydrogel promotes angiogenesis during vascularized bone regeneration. ACS Appl Mater Interfaces 2020;12:22410–25. 32349479 10.1021/acsami.0c00714

[37] Pan S , Yin Z, Shi C, Xiu H, Wu G, Heng Y, Zhu Z, Zhang J, Gui J, Yu Z, Liang B. Multifunctional injectable hydrogel microparticles loaded with miR-29a abundant BMSCs derived exosomes enhanced bone regeneration by regulating osteogenesis and angiogenesis. Small 2023;20:e2306721. 38018340 10.1002/smll.202306721

[38] Huang Y , Du Z, Li K, Jing W, Wei P, Zhao B, Yu Y, Cai Q, Yang X. ROS-scavenging electroactive polyphosphazene-based core–shell nanofibers for bone regeneration. Adv Fiber Mater 2022;4:894–907.

[39] Shen Y , Jiang B, Luo B, Jiang X, Zhang Y, Wang Q. Circular RNA-FK501 binding protein 51 boosts bone marrow mesenchymal stem cell proliferation and osteogenic differentiation via modulating microRNA-205-5p/Runt-associated transcription factor 2 axis. J Orthop Surg Res 2023;18:782. 37853466 10.1186/s13018-023-04242-1 PMC10583363

[40] Vimalraj S. Alkaline phosphatase: structure, expression and its function in bone mineralization. Gene 2020;754:144855. 32522695 10.1016/j.gene.2020.144855

[41] Kihara T , Hirose M, Oshima A, Ohgushi H. Exogenous type I collagen facilitates osteogenic differentiation and acts as a substrate for mineralization of rat marrow mesenchymal stem cells in vitro. Biochem Biophys Res Commun 2006;341:1029–35. 16458256 10.1016/j.bbrc.2006.01.059

[42] Gordon JA , Tye CE, Sampaio AV, Underhill TM, Hunter GK, Goldberg HA. Bone sialoprotein expression enhances osteoblast differentiation and matrix mineralization in vitro. Bone 2007;41:462–73. 17572166 10.1016/j.bone.2007.04.191

[43] Carvalho MS , Silva JC, Hoff CM, Cabral JMS, Linhardt RJ, da Silva CL, Vashishth D. Loss and rescue of osteocalcin and osteopontin modulate osteogenic and angiogenic features of mesenchymal stem/stromal cells. J Cell Physiol 2020;235:7496–515. 32162324 10.1002/jcp.29653

[44] Leucht P , Lee S, Yim N. Wnt signaling and bone regeneration: can’t have one without the other. Biomaterials 2019;196:46–50. 29573821 10.1016/j.biomaterials.2018.03.029

[45] Vermeulen S , Tahmasebi Birgani Z, Habibovic P. Biomaterial-induced pathway modulation for bone regeneration. Biomaterials 2022;283:121431. 35231787 10.1016/j.biomaterials.2022.121431

[46] Yu H , Zhu D, Liu P, Yang Q, Gao J, Huang Y, Chen Y, Gao Y, Zhang C. Osthole stimulates bone formation, drives vascularization and retards adipogenesis to alleviate alcohol-induced osteonecrosis of the femoral head. J Cell Mol Med 2020;24:4439–51. 32135036 10.1111/jcmm.15103 PMC7176840

[47] Ucuzian AA , Gassman AA, East AT, Greisler HP. Molecular mediators of angiogenesis. J Burn Care Res 2010;31:158–75. 20061852 10.1097/BCR.0b013e3181c7ed82 PMC2818794

[48] Zhai Y , Schilling K, Wang T, El Khatib M, Vinogradov S, Brown EB, Zhang X. Spatiotemporal blood vessel specification at the osteogenesis and angiogenesis interface of biomimetic nanofiber-enabled bone tissue engineering. Biomaterials 2021;276:121041. 34343857 10.1016/j.biomaterials.2021.121041 PMC8477312

[49] Rahbarghazi R , Nassiri SM, Khazraiinia P, Kajbafzadeh AM, Ahmadi SH, Mohammadi E, Molazem M, Zamani-Ahmadmahmudi M. Juxtacrine and paracrine interactions of rat marrow-derived mesenchymal stem cells, muscle-derived satellite cells, and neonatal cardiomyocytes with endothelial cells in angiogenesis dynamics. Stem Cells Dev 2013;22:855–65. 23072248 10.1089/scd.2012.0377 PMC3585743

[50] Charbord P. Bone marrow mesenchymal stem cells: historical overview and concepts. Hum Gene Ther 2010;21:1045–56. 20565251 10.1089/hum.2010.115 PMC4823383

[51] Xu X , Xiao L, Xu Y, Zhuo J, Yang X, Li L, Xiao N, Tao J, Zhong Q, Li Y, Chen Y, Du Z, Luo K. Vascularized bone regeneration accelerated by 3D-printed nanosilicate-functionalized polycaprolactone scaffold. Regen Biomater 2021;8:rbab061. 34858634 10.1093/rb/rbab061 PMC8633727

[52] Qazi TH , Mooney DJ, Duda GN, Geissler S. Biomaterials that promote cell-cell interactions enhance the paracrine function of MSCs. Biomaterials 2017;140:103–14. 28644976 10.1016/j.biomaterials.2017.06.019

[53] Duan J , Lei D, Ling C, Wang Y, Cao Z, Zhang M, Zhang H, You Z, Yao Q. Three-dimensional-printed polycaprolactone scaffolds with interconnected hollow-pipe structures for enhanced bone regeneration. Regen Biomater 2022;9:rbac033. 35719204 10.1093/rb/rbac033 PMC9201971

[54] Gallagher LB , Dolan EB, O'Sullivan J, Levey R, Cavanagh BL, Kovarova L, Pravda M, Velebny V, Farrell T, O'Brien FJ, Duffy GP. Pre-culture of mesenchymal stem cells within RGD-modified hyaluronic acid hydrogel improves their resilience to ischaemic conditions. Acta Biomater 2020;107:78–90. 32145393 10.1016/j.actbio.2020.02.043

[55] Wang J , Li W, Lu Z, Zhang L, Hu Y, Li Q, Du W, Feng X, Jia H, Liu BF. The use of RGD-engineered exosomes for enhanced targeting ability and synergistic therapy toward angiogenesis. Nanoscale 2017;9:15598–605. 28990632 10.1039/c7nr04425a

[56] Liu G , Chen J, Wang X, Liu Y, Ma Y, Tu X. Functionalized 3D-printed ST2/gelatin methacryloyl/polcaprolactone scaffolds for enhancing bone regeneration with vascularization. Int J Mol Sci 2022;23:8347. 35955478 10.3390/ijms23158347 PMC9368581

[57] Wang L , Pang Y, Tang Y, Wang X, Zhang D, Zhang X, Yu Y, Yang X, Cai Q. A biomimetic piezoelectric scaffold with sustained Mg(2+) release promotes neurogenic and angiogenic differentiation for enhanced bone regeneration. Bioact Mater 2023;25:399–414. 37056250 10.1016/j.bioactmat.2022.11.004 PMC10087109

[58] Li Y , Zhang J, Chen L, Li H, Wang J. Repair of critical-sized rat cranial defects with RADA16-W9 self-assembled peptide hydrogel. Biochem Biophys Res Commun 2023;652:68–75. 36812709 10.1016/j.bbrc.2023.02.028

[59] Lertkiatmongkol P , Liao D, Mei H, Hu Y, Newman PJ. Endothelial functions of platelet/endothelial cell adhesion molecule-1 (CD31). Curr Opin Hematol 2016;23:253–9. 27055047 10.1097/MOH.0000000000000239 PMC4986701

[60] Morgan SM , Samulowitz U, Darley L, Simmons DL, Vestweber D. Biochemical characterization and molecular cloning of a novel endothelial-specific sialomucin. Blood 1999;93:165–75. 9864158

[61] Xu Z , Kusumbe AP, Cai H, Wan Q, Chen J. Type H blood vessels in coupling angiogenesis-osteogenesis and its application in bone tissue engineering. J Biomed Mater Res B Appl Biomater 2023;111:1434–46. 36880538 10.1002/jbm.b.35243

[62] Qiu M , Li C, Cai Z, Li C, Yang K, Tulufu N, Chen B, Cheng L, Zhuang C, Liu Z, Qi J, Cui W, Deng L. 3D biomimetic calcified cartilaginous callus that induces type H vessels formation and osteoclastogenesis. Adv Sci (Weinh) 2023;10:e2207089. 36999832 10.1002/advs.202207089 PMC10238192

