# Supplementary material for: Self-assembled peptide hydrogel loaded with functional peptide Dentonin accelerates vascularized bone tissue regeneration in critical-size bone defects
Source: Regen Biomater. 2024 Aug 23;11:rbae106. doi: 10.1093/rb/rbae106 (PMC11387769; doi:10.1093/rb/rbae106)
Supplement: rbae106_Supplementary_Data [file rbae106_supplementary_data.docx]

**Supplementary Information**

**Self-assembled peptide hydrogel loaded with functional peptide Dentonin accelerates vascularized bone tissue regeneration in critical-size bone defects**

Yijuan Liu^1,2^†, Li Li ^1,2^†, Mengjiao He^1,2^, Yanmei Xu^1,2^, Zekai Wu^1,2^, Xiongcheng Xu^1,2^, Luo Kai^1,2^*, Hongbing Lv^1,2^*

^1^ Fujian Key Laboratory of Oral Diseases & Fujian Provincial Engineering Research Center of Oral Biomaterial & Stomatological Key Laboratory of Fujian College and University, School and Hospital of Stomatology, Fujian Medical University, Fuzhou, People’s Republic of China.

^2^ Institute of Stomatology & Laboratory of Oral Tissue Engineering, School and Hospital of Stomatology, Fujian Medical University, Fuzhou 350002, People’s Republic of China.

† The authors contribute equally to this work.

*Correspondence: Luo Kai; Hongbing Lv

School and Hospital of Stomatology, Fujian Medical University, Fuzhou, People’s Republic of China.

E-mail : luokai39@163.com ; hongbinglu@126.com

## Results

| **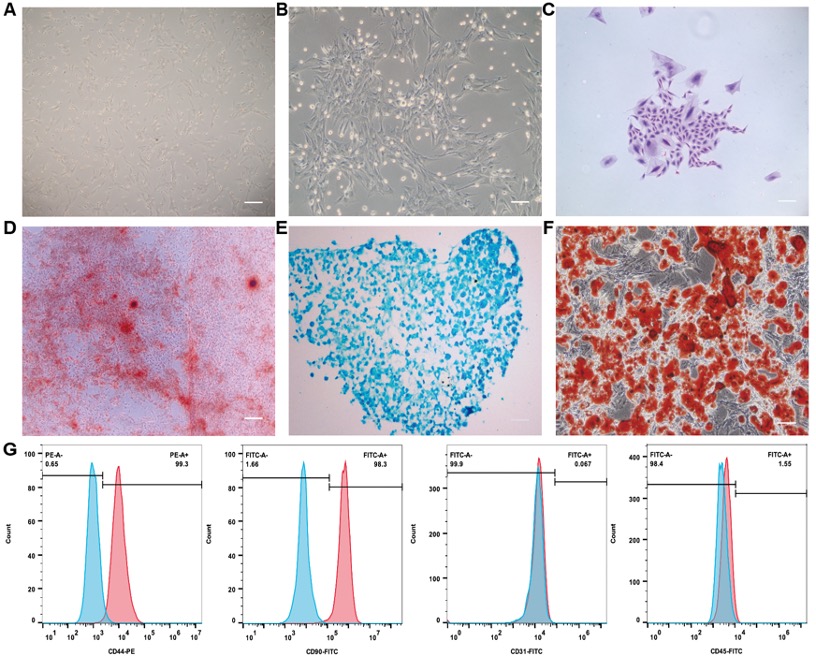** |
| --- |
| **Figure. S1.** Culture and characterization of rat BMSCs. (A) Primary BMSCs and (B) first-generation BMSCs after subculturing. (C) Crystal violet staining to observe the colony-forming ability of BMSCs; scale bar: 200 μm. (D) BMSCs formed mineralized nodules in induction medium (Alizarin Red S, 200 μm), (E) acidic mucopolysaccharide (Alcian Blue, 100 μm) and (F) lipid-rich vacuoles (Oil Red O, 100 μm). (G) Flow cytometric analysis of the immunophenotypic expression of BMSCs, showing positive expression of CD44 (99.3%) and CD90 (98.3%), and negative expression of CD31 (0.067%) and CD45 (1.55%). |
